# Cigarette Smoking in Indonesia: Examination of a Myopic Model of Addictive Behaviour

**DOI:** 10.3390/ijerph7062473

**Published:** 2010-06-04

**Authors:** Budi Hidayat, Hasbullah Thabrany

**Affiliations:** 1 Faculty of Public Health, the University of Indonesia, Kampus FKM UI, Building F 1th Floor, Depok 16424, Indonesia; 2 Poverty Reduction and Economic Management, the World Bank Office Jakarta, Jakarta Stock Exchange Building Tower 2, 12th Floor, Jl. Jend. Sudirman Kav. 52–53, Jakarta 12190, Indonesia; 3 Center for Health Economic and Policy Analyses Studies, Faculty of Public Health, the University of Indonesia, Kampus FKM UI, Building G 3th Floor, Depok 16424, Indonesia; E-Mail: hasbullah.thabrany@ui.edu

**Keywords:** cigarette consumption, myopic addictive models, methodology for panel data, Indonesia

## Abstract

Using aggregated panel data taken from three waves of the Indonesian Family Life Survey (1993–2000), this article tests the myopic addiction behaviour of cigarette demand. Sensitivity analysis is done by examining a rational addiction behavior of cigarette demand. The results provide support for myopic addiction. The short- and long-run price elasticities of cigarette demand are estimated at −0.28 and −0.73 respectively. Excise taxes are more likely to act as an effective tobacco control in the long-run rather than a major source of government revenue.

## 1. Introduction

Economic models of addiction can be divided into three groups: imperfectly rational addiction, rational addiction and myopic addiction [1]. The last assumes individuals recognize the dependence of current addictive good consumption on past consumption, but ignore the impact of current and past choices on future consumption decisions when making current choices. Researchers investigating myopic addiction always build consumption history into models, but ignore anticipated future changes. There are a number of studies investigating myopic addiction in both pooled and time series frameworks. Empirical applications of myopic addiction in cigarette consumption include, *inter alia*, Baltagi and Levin [2,3]. Alternative dynamic specifications of addictive cigarette consumption are found in Cameron’s survey [4].

Indonesia is a significant contributor to the global burden of disease from tobacco-related illnesses. The prevalence of smoking among males age 15 years and above increased from 53.4% in 1995 to 63.2% in 2001 and to 63.1% in 2004. Among adult females, the prevalence of smoking has also increased from 1.7% in 1995 to 4.5% in 2004. Overall, cigarettes consumed increased from 33 billion in 1970 to 217 billion in 2004. With the fourth largest population in the world, Indonesia in 2002 ranked as the fifth largest consumers of cigarettes (182 billion) behind China (1.7 trillion), USA (463 billion), Russia (375 billion), and Japan (299 billion) [5]. A study by Djutaharta *et al.* [6] reported that 90 percent of active smokers in Indonesia smoked in their house when other family members, including children, were around; as a result, the children have higher incidences of pulmonary diseases.

Increasing the price of tobacco is one of several strategies used to curb tobacco consumption [7]. Evidence from high-income countries shows that increases in cigarette (and other tobacco products) taxes are often followed by significant reductions in cigarette smoking (and other tobacco use). These changes reflect a combination of increased smoking cessation, reduced rates of relapse and initiation, and decreased consumption among continuing tobacco users. Estimates of the price elasticity of cigarette demand in high-income countries range from −0.25 to −0.50 while estimates from low- and middle-income countries are approximately double: −0.50 to −1.00 [7]. The evidence and estimates suggest that tobacco taxes in low-income countries could be effective in reducing tobacco use. Additionally, as children and adolescents are often more responsive than adults to prices changes, these groups may account for a relatively larger portion of overall reductions or a relatively faster response or both. For health policy in emerging middle-income Indonesia, then, it is important to understand if increased taxes are likely to have a significant impact on tobacco consumption in an environment where both incomes and smoking behavior are on the rise.

Evidence on tobacco consumption behavior from Indonesia is rare. A prior study using data from the 1999 national socio-economic survey [8] did not model the long-run dynamics of cigarette demand but did provide estimates of short-run elasticity. Conditional on tobacco product use, demand price elasticity was found to be −0.6, and this decreased (in absolute terms) with income.

There have been no complete estimates for Indonesia of demand response to changes in taxes on tobacco products. These estimates (short-run and long-run) are crucial for both health and revenue policy. Furthermore, understanding addictive behavior in developing countries is an important regulatory question since the tobacco industry has focused on the developing world as a target for new customers. To the best of the authors’ knowledge, there is no study that estimates for Indonesia a dynamic specification (including addictive behavior) of cigarette demand with complete information on demand price elasticities. This paper will investigate such a model for cigarette demand and use the model to estimate both short- and long-run price elasticities of cigarette demand in Indonesia.

The addiction model developed here is tested using pooled individual panel data from three waves of the Indonesian Family Life Survey (IFLS) spanning the period 1993–2000. Although aggregate panels have less variability relative to individual level data, and this has been regarded as a weakness of time series studies [9], the use of panel data actually provides two advantages. First, we can control for unobserved heterogeneity at the individual level that otherwise might confound results. Second, it enables tests of the dynamics of cigarette demand necessary for estimates of long-run demand price elasticity.

Most of the existing studies on price elasticities in developing countries do not develop model dynamics that contain rational addiction. Studies that develop myopic addiction include Tansel [10] for Turkey, Hsieh and Hu [11] for Taiwan, Van Walbeek [12] for South Africa and Jamaica and Guindon *et al.* [13] for Southeast Asian countries. The study here adds empirical evidence to myopic addiction models of cigarette demand and also provides a sensitivity analysis by estimating a model of rational addiction with the same data. Note that dynamic models with addictive behavior predict long-run demand price elasticities will be larger than short-run elasticities (in absolute value). We therefore anticipate unusually low short-run elasticities derived from our models. We explore several estimators for panel data and develop a framework for selecting the best estimator. Documenting this econometric selection process is important for transparency in research and also for enhancing techniques among like-minded practicioners.

## 2. Methodology

In this study, we estimate a myopic addiction for cigarette demand model in the form:

(1) $$C_{it}=\beta_{0}+\beta_{1}C_{it-1}+\beta_{2}Pc_{it}+\beta_{3}Pa_{it}+\beta_{4}{x^{\prime }}_{it}+\upsilon_{i}+d_{t}+\varepsilon_{it}$$

where *i* is an individual, *t* is time, *C* is consumption of cigarettes, *Pc* and *Pa* are prices of cigarettes and alcohol, respectively, *x*′ is a vector of exogenous variable that affect consumption of cigarettes including disposable income, age, employment status, and the presence of children less than 14 years of age, *v_i_* is an individual fixed effect controlling for time-invariant preferences and marginal utility of wealth), *d_t_* is a time dummy controlling for unanticipated macro changes in wealth, and ε*_it_* is the error term. A significant and positive effect of previous consumption (measured by coefficient *β*_1_) on current cigarette consumption (*C_it_*) indicates myopic addictive behavior [14].

We also perform a sensitivity analysis that examines myopic versus rational addiction. Empirical specifications for rational addiction are as follows:

(2) $$C_{it}=\beta_{0}+\beta_{1}C_{it-1}+\beta_{2}C_{it+1}+\beta_{3}Pc_{it}+\beta_{4}Pa_{it}+\beta_{5}{x^{\prime }}_{it}+\upsilon_{i}+d_{t}+\varepsilon_{it}$$

Statistical significance of the coefficient (*β*_2_) on lead consumption (Equation 2) together with a reasonable estimate of the discount rate gives a direct test of a rational addiction model against an alternative model in which consumers are myopic [14–16].

Applying ordinary least squares (OLS) in Equations (1) and (2) could lead to biased parameter estimates for at least two reasons. First, the errors *ɛ_it_* may be serially correlated with and through lagged and lead consumption. That is, there could be an omitted variable bias from unobserved time-invariant preferences, marginal utility of wealth (*v_i_*) and other demand shifters (*e_it_*) that may be serially correlated. These unmeasured variables may be correlated with *C_it_*_−1_ in Equation (1) and both *C_it_*_−1_ and *C_it+_*_1_ in Equation (2). Second, there is measurement error in recorded values of *C_it_*_−_*_1_* in Equation (1) and both *C_it_*_−1_ and *C_it_*_+1_ in Equation (2). Equations (1) and (2) were derived assuming perfect certainty on prices and other variables; when unexpected changes in these variables cause individuals to revise their consumption plans, *C_it_*_−1_ and *C_it_*_+1_ then measured with error. Measurement error in either dependent or independent variables leads to biased OLS coefficient estimates.

We explored two groups of econometric specifications in our analysis. The first group includes estimators ignoring the endogenous-regressors problem, including OLS, fixed effects (FE), and random effects (RE). The second group includes estimators that treat error-in-variables and unobservable heterogeneity: two-stage least squares (2SLS), fixed effects two-stage least squares (FE2SLS), random effects two-stage least squares (RE2SLS), and generalized methods of moments (GMM).

Diagnostic tests helped us select the most appropriate estimator. We tested for endogeneity in lagged consumption by performing a Hausman specification test. If lagged consumption is found to be exogenous, we opt for either OLS, FE or RE. To test the appropriateness of an individual-effects estimator instead of regular OLS, we use the Breusch-Pagan Lagrange Multiplier test [17]. If the test suggests that time-invariant unobserved characteristics are affecting choices random or fixed effects is called for. The choice between FE and RE relies on comparison of the two estimators in Hausman’s specification test. Once the null hypothesis of no correlation between the individual effect and the in Equation (1) is rejected, then we opt to use FE [17].

However, if lagged consumption is indeed endogenous, we suggest instruments (and maintain that they are valid) and use estimators from Group II. To evaluate whether there may be a bias from weak instruments, first we run the OLS regressions for the lagged consumption *C_it_*_−1_:

(3) $$C_{it-1}=\beta_{0}+\beta_{1}z_{i}+\beta_{2}Pc_{it}+\beta_{3}Pa_{it}+\beta_{4}{x^{\prime }}_{it}+\upsilon_{i}+d_{t}+\varepsilon_{it}$$

where *z_i_* are the potential instruments and all else is as defined in Equation (1). The instruments will be valid if they are good predictors of lagged consumption and uncorrelated with the error in the demand Equation (1). In addition to checking that the instruments are significant in the first stage regression (3), we performed several tests of the instruments, including relevancy, validity and orthogonality. Recent applications of these tests and the selection process to choose the best estimator are described elsewhere [18].

## 3. Data and Variables

The Indonesian Family Life Survey (IFLS) was carried out by the RAND Corporation in conjunction with Indonesian researchers and various international agencies in 1993 (IFLS1), 1997 (IFLS2) and in 2000 (IFLS3). IFLS is a panel and the sampling scheme for the first wave (IFLS1) is determines the sample in subsequent waves. IFLS1 sampling is stratified on provinces and urban/rural location, then randomly sampled within these strata. The IFLS sample included 13 of Indonesia’s 26 provinces which contained 83% of the 1993 population. Within each of the 13 provinces, enumeration areas (EAs) were randomly chosen from a nationally representative sample frame used in the 1993 national social economic survey (SUSENAS), yielding 321 EAs with oversampling in urban areas and in smaller provinces to facilitate urban-rural and Javanese–non-Javanese comparisons. Within an EA households were randomly selected based upon 1993 SUSENAS listings and twenty (thirty) households were selected from each urban (rural) EA.

IFLS1 contacted a total of 7,730 households and obtained a final sample size of 7,224 households. In IFLS2 6751 of the original 7,224 households (93.5%) were relocated and re-interviewed. In IFLS3 the re-contact rate was 95.3% of IFLS1 households. Nearly 91% of households are complete panel households interviewed in all three waves. IFLS contains measures of smoking behavior from individuals aged 15 and above. Frankenberg and Karoly [19], Frankenberg and Thomas [20], and Straus *et al*. [21] described more fully IFLS1, IFLS2 and IFLS3, respectively.

Table 1 gives a description and corresponding summary statistics of the variables used here. We measure cigarette consumption (the dependent variable) as the number of cigarettes per day smoked as recalled by the individual at the time of the interview. We constructed this variable from three questions: (i) “*have you had the habit of chewing tobacco, smoking a pipe, smoking self-rolled cigarettes, or smoking cigarettes/cigars*?” (ii) “*do you still have the habit or have you totally quit*?, and (iii) “*in one day about how many cigars/cigarettes did you consume now/before totally quitting*?”. The variable takes the value zero when the individual is an ex- or non-smoker.

Explanatory variables include the number of cigarettes smoked as recorded in previous (lag) waves, which we take as a measure of the effects of past cigarette consumption on current marginal utility of cigarette consumption. For testing the rational addiction model we also included the number of cigarettes smoked in the next (lead) wave.

We included measures of cigarette and alcohol prices at the time of the interview. The IFLS formats these questions differently across waves. For IFLS2 and IFLS3, cigarette prices were constructed from individual responses to the following question: “*about how much money did/do you spend each week for tobacco products*?” In IFLS1, cigarette prices were derived from household expenditure information. Real prices were calculated from these nominal prices by using consumer price index (CPI) data from the Central Bureau of Statistics.

Other time-varying explanatory variables included a monthly income proxy (from expenditure recall data), expressed as a real value with 2,000 CPI data. To obtain a per-equivalent adult measure of consumption, all income proxy data was adjusted for family size. The natural log of age was included to control for age-related health problems from smoking. A dummy variable indicating the presence of children less than 14 years of age was also included; we assumed that individuals might moderate tobacco consumption when small children are present. We also included a dummy variables indicating employment status.

For Group II estimators (discussed above) an instrument is needed. Appropriate instrumental variables in our context will play an important role in determining past cigarette consumption (a potentially endogenous variable) but will not affect current consumption (the dependent variable) except through past consumption. For the myopic model, we instrumented the lagged consumption with lagged cigarette prices. Other dummy variables, which we consider to be proxies for wealth or economic stability, were also included as potential instruments for lagged consumption: dwelling walls are brick; dwelling floor is permanent; dwelling is owned or being bought (1/0); and individual’s religion.

For the rational addiction model, we also included one lead of tobacco price as an instrument for future cigarette consumption. We avoided lagged values of cigarette consumption as instruments for lead consumption due to concerns about serial correlation in the errors.

## 4. Results

### 4.1. Model Selections

We first checked for endogeneity of the lagged consumption variable in the demand equation using Durbin-Wu Hausman and Hausman-Wu statistical tests. This is a Likelihood Ratio (LR) test distributed as a *x*^2^ with 1 degree of freedom. The value of the test was about 7.9 with a p-value of 0.005. We thus rejected the null hypothesis of exogeneity (Table 2), suggesting OLS results in inconsistent parameter estimates [17].

A further consideration is to choose estimators among the alternatives in Group II. The Pagan and Hall’s test [22] for heteroskedasticity were adopted to discriminate between 2SLS and GMM estimators. The test rejected the null hypothesis of homoskedasticity, suggesting GMM is preferable to 2SLS. We then considered between random effects 2SLS and fixed effect 2SLS. The resulting Hausman statistic test yielded an observed chi-squared test of 12.9, and was insignificant at 5 percent level. We therefore did not reject the null hypothesis of no correlation between the individual effect and the *x*′*_it_* in Equation (1), suggesting the RE2SLS is preferable than the FE2SLS.

A number of tests were employed to test the relevancy, validity and orthogonality requirements of the instruments. A reduced form regression of the suspected endogenous variable, the lagged consumption, on the full set of instruments was estimated using OLS. The coefficients on the instruments in first-stage least squares *C_it_*_−1_ equations are given in Table 3.

The *R*^2^ shows that the models explained a high proportion (12 percent) of the variation for lagged consumption. Table 3 also reports the Partial *R*^2^ and Shea Partial *R*^2^. A gap between the Partial *R*^2^ and Shea partial *R*^2^ in our study considerably a small, suggesting the model is well-identified [23]. The relevance of the instruments was also investigated using an *F*-test to determine whether the instruments were correlated with the potentially endogenous variable [24,25]. The null hypothesis of the *F*-test that the parameters of the covariates are jointly equal to zero was rejected, indicating that all the instruments were jointly significant (see, the last row of Table 3). A conservative rule of thumb for a single endogenous regressor would suggest that a less than 10 *F*-value could be an indicator of a weak instrument [23]. In this study, the *F*-test for all instruments and for five instruments yielded 61 and 18, respectively.

The proposed instruments also passed the over-identification tests. The Hansen-*J*, Basmann and Sargan statistic tests (Table 2) could not reject the null hypothesis of correct specification, suggesting the models are reasonably well specified and the instruments are valid. Finally, the orthogonality condition of the instruments assessed using the *C*- test (Table 2). The *C*-test could not reject the null hypothesis, indicating the subset instruments used, the lagged prices, are exogenous.

### 4.2. Model Estimation Results

Table 4 presents the GMM estimation results of both myopic and rational addiction model. Coefficient estimate of lagged smoking in Equations (1) and (2) was 0.625 and 0.521, respectively, and significant at 1 percent level. This finding suggests that cigarette is an addictive good, and myopic addiction hypothesis is accepted. Estimate coefficient of lead smoking in Equation (2) was smaller than the coefficients of lagged smoking (0.112 *vs*. 0.509). This finding is consistent with the theory, which rises to positive rate and reasonable time preference. Given the lead consumption turned out to be insignificant, the rational addiction hypothesis was rejected in favor of the myopic one.

The effect of price was always significant at 1 percent level. Cigarette prices had a negative effect on smoking, whilst alcohol prices had a positive and significant effect on smoking, suggesting that alcohol and cigarette are substitutes. Although cigarette consumption was found to be positively associated with income, the finding was insignificant. The low and insignificant income elasticity here (0.015) is not uncommon in the contexts of pooled models and developing countries. Blecher [26] found very low income elasticities in developing countries vis-à-vis developed countries although it used aggregate data. Coefficient estimate on children aged ≤14 turned out to be a positive, although it was insignificant, suggesting smokers did not reduce or attempt to moderate cigarettes consumption although they had small children. As expected individuals having working status increased the number of current cigarettes consumption and was significant at the 1 percent level.

The last row of the Table 4 presents short- and long-run price elasticity of demand. Coefficient estimate of cigarette price indicates the short-run price elasticity of demand. The long-run one in equation 1 was computed as: *∂E*(*LnC_it_*)/ *∂E*(*LnPc_it_*) = *β̂*_2_ / (1–*β̂*_1_). The short-run and long-run price elasticities, evaluated at the mean, were −0.28 and −0.73, respectively. The findings that the long-run price elasticity, in absolute value, exceeds the short-run one is in line with both theoretical expectations and empirical findings. For Equation 2, the long run price elasticity is calculated using the expression *∂E*(*LnC_it_*)/ *∂E*(*LnP_it_*) = *β̂*_3_ /(1–*β̂*_1_ – *β̂*_2_), and the implied discount factor and the discount rate: *β_2_/β_1_*, *β_1_/β_2_*-1, respectively.

## 5. Discussion

This study aimed to investigate the demand for cigarettes through a myopic addiction model and to use this model to estimate the price elasticity of cigarette demand in Indonesia. Sensitivity analysis was done by examining a rational addiction model. We explored several empirical approaches using panel data and selected the most appropriate techniques given both endogeneity of regressors and behavior of the error terms (*i.e*., the correlation between individuals effects, ε*_it_*, and the regressors, *x*′*_it_*).

Results suggest that variables we suspected might be endogenous are indeed endogenous in our model. The Group II estimators are appropriate for handling endogeneity. To select among the four alternatives, we adopted the Pagan and Hall statistic tests for testing unknown heteroskedasticity. Since the use of either a random- or fixed-effects model can be justified by a Wu-Hausman test, we used this test to assess whether regression parameters characterizing the random outcome variable stay constant across all cross-sectional units for all time periods [28]. Because the homogeneity hypothesis was rejected, we conclude that GMM is the best estimator to handle unknown heteroskedasticity. Either random- or fixed-effects may suffer from heteroskedasticity (non-constant variance in and/or serial correlation) [28]. Applying 2SLS also would lead to invalid inference as standard errors are inconsistent in the presence of unknown heteroskedasticity [27].

We confirm that Indonesian smokers are myopic addicts. Estimates yield a positive coefficient on lagged consumption that is highly statistically significant in both Equations (1) and (2). Our findings imply that higher past consumption raises the marginal utility of current consumption and leads to higher current consumption. Lagged consumption represents a fixed addictive tendency carried over from period to period and its coefficient can be interpreted as the speed of adjustment to steady-state consumption. Adjustment to any new steady-state consumption level takes place in more than one period following a cigarette price change, but there is also an immediate response given by the impact multiplier. This study finds that long-run cigarette price effects (or equilibrium multiplier) exceeded the short-run effects. This finding is in line with both theoretical expectations and previous empirical findings. Analyses from various Southeast Asian countries have found that the short-run price elasticity estimates for tobacco products range from −0.17 to −0.78, while the long-run estimates range from −0.4 to −1.21 [13].

Price increases had a negative and significant impact on cigarette consumption. The elasticity estimates suggest that a 10 percent increase in cigarette prices would lead to a 2.8 percent decrease in cigarette consumption in the short run and 7.3 percent decreases in the long run. Although our elasticity estimates seem low given other developing country estimates, they are comparable to other pooled data studies from developed countries. Pooled data studies have shown elasticities which are consistently lower than those in time series studies. The estimates from pooled data of the impact of price on cigarette consumption in developed countries (Baltagi and Levin [2], Stewart [29], and Sung *et al*. [30]) are at the very low end of those from time series studies, from −0.22 to 0.50.

The effect of cigarette price on consumption has important implications for revenue and health policies. Increasing cigarette prices via excise taxes can control tobacco use and at the same time raise government revenue. Our study finds cigarette demand is inelastic (with a price elasticity less than one), suggesting the percentage increase in prices would always be larger than the consumption response. However, we also estimate demand to be more elastic in the long-run than in the short-run. A long-run price elasticity of −0.73 indicates that tobacco taxation can be an effective tool to reduce cigarette consumption for Indonesia. Empirical studies have shown that tax increases are regarded as the most effective tool for tobacco consumption reduction and have been especially effective among young people and people with low incomes [7].

Should taxes be calibrated to be revenue maximizing or calibrated to maximize the reduction in tobacco consumption? Addiction in our study is a combination of habit and preference adaptation, starting with subjective individual preferences: individual smokers reveal that they gain net utility (or satisfaction) from tobacco consumption. For public health priorities, our model indicates that “addiction” can be conditioned and does respond to incentives. So for both revenue and health policymakers, we provide a methodological innovation for analyzing how tobacco tax policy can maximize revenue given public health goal or *vice versa*.

Our elasticity estimates suggests that price increases brought about by higher taxes would cause government revenue to increase as the proportionate change in prices would exceed the proportionate change in consumption. However, if is thought that tax policy exploits true addicts, whose demand elasticity is very high, to compensate for revenues lost to decreases in consumption among smokers who are only addicted in the technical economist’s sense, then optimal tax rates would tend to be underestimated by our economic models. Our models also may be insensitive to heterogeneity in the population of smokers with respect to elasticities. Thus, improved estimates of price sensitivity at different income levels and knowledge of the extent to which tobacco users underestimate health harm in the Indonesia context can better inform tobacco control efforts.

The tax revenue generated from this policy could logically be used to increase public health budgets as smokers impart negative externalities to non-smokers and to public health. However, given that the demand for cigarettes is more elastic in the long-run, further excise tax increases are more likely to act as a tobacco control mechanism in the long-run rather than as a constant source of government revenue. Future increase in the tax will induce some smokers to quit and prevent others from becoming regular or persistent smokers. They also will reduce the number of ex-smokers returning to cigarettes and will reduce consumption among continuing smokers. Empirical evidence from South Africa shows that a doubling of the real price of cigarettes between 1993 and 2003 would reduce consumption by a quarter in the short term [12]. These gains would be significant in South Africa or any other country struggling with the public health consequences of high rates of tobacco consumption.

The effects of the price of alcohol call for further research. Higher alcohol prices lead to higher cigarette consumption in our empirical specifications. This positive cross-elasticity could indicate that alcohol and cigarettes are substitutes, which might contradict evidence from developed countries for their complementarity. Our finding may be due to a lower proportion of true addicts among a low- income population. We are unable to test this assumption within our models and empirical specifications. Another natural concern is other tobacco-related substitutes. If demand for cigarettes falls, does this mean that the demand for other tobacco products will increase? Future research should gather information about potential substitution of other tobacco products, such as kreteks, bidis, and hand rolled cigarettes for manufactured cigarettes. These substitution issues have an obvious bearing on tobacco control policy and its effectiveness.

## Figures and Tables

**Table 1.** Definition of variables used in the models and its descriptive statistic.

**Variable**	**Definition**	**Mean**	**Std. Dev.**
*C_t_*	Current cigarette consumption (ln)	2.207	0.746
*C_it_*_−1_	One lag cigarette consumption (ln)	2.203	0.760
*Pc_t_*	Current price cigarette (ln)	4.623	0.701
*Pa_t_*	Current price alcohol (ln)	8.612	1.263
Ln-exp	Monthly per-capita income (ln)	11.156	1.004
Working	1 if working, 0 otherwise	0.582	0.493
Ln-age	Individual age (ln)		
Child14	1 if children aged ≤14, 0 otherwise		
Instruments (z)			
*Pc_t_*_−1_	One lag price cigarette (ln)	4.280	0.520
Wall	1 if dwelling wall is brick, 0 otherwise	0.588	0.492
Floor	1 if dwelling floor is permanent, 0 otherwise	0.155	0.362
Hhown	1 if dwelling is owned/bought, 0 otherwise	0.805	0.396
Moslem	1 if Moslem, 0 otherwise	0.871	0.335

**Table 2.** Summary statistics test: endogeneity and instrumental tests.

**Test statistics**	**Statistics**
**1. Endogeneity of** lagged *C_it_*_−1_	
Wu-Hausman	7.91^***^
Durbin Wu Hausman (DWH): *x*^2^(1)	7.91^***^
**2. Instrumental variable:**	
a. Heteroskedasticity*: x*^2^(11)	
Pagan-Hall general test statistic	66.52^***^
Pagan-Hall test with assumed normality	125.30^***^
White/Koenker n*R*^2^ test statistic	75.57^***^
Breusch-Pagan/Godfrey/Cook-Weisberg	148.77^***^
b. Overidentifying:	
Sargan (2SLS)	4.03
Basmann (2SLS)	4.03
Hansen-*J* (GMM)	4.01
c. Orthogonality:	
*C*-statistics: *x*^2^(1)	0.37

**Table 3.** First-stage regression of the lagged (***C*_*it*−1_**): OLS estimates.

	Coef.	SE
Pc_t_	−0.0559^***^	0.020
Pa_t_	0.2253^***^	0.012
Ln-exp	−0.027**	0.014
Ln-age	0.3470^***^	0.031
If Child14	0.2775^***^	0.050
If working	0.0995^***^	0.027
Excluded instruments:		
*Pc_t_*_−_*_1_*	−0.0033	0.022
If dwelling wall is brick	−0.0976^***^	0.020
If dwelling floor is permanent	−0.1715^***^	0.029
If dwelling is owned or being bought	−0.0744^***^	0.024
If Moslem	−0.1404^***^	0.029
Constant	−0.3265	0.213

*R*^2^	0.121	
Shea partial *R*^2^	0.016	
Partial *R*^2^	0.016	
Test of *F*:		
All instruments, F(11, 4107)	61.04^†^	
Excluded instruments, F(5, 4107)	18.04^†^	

Significance at 1% level and

** 5%; SE is robust standard errors.

**Table 4.** GMM estimation results: myopic vs. rational addiction models.

	**Myopic Addiction Model [Equation (1)]**	**Rational Addiction Model [Equation (2)]**
Lagged consumption (*C_t−1_*)	0.625*** [0.081]	0.509*** [0.117]
Lead consumption (*C_t+1_*)	n.a n.a	0.112 [0.157]
Price cigarette (*Pc_t_*)	−0.275*** [0.019]	−0.135** [0.066]
Price alcohol (*Pa_t_*)	0.143*** [0.021]	0.159*** [0.033]
Per–capita income (Ln)	0.015 [0.012]	−0.01 [0.019]
Individual age (Ln)	−0.098*** [0.038]	0.053 [0.063]
If child14 exist	0.048 [0.044]	0.097 [0.071]
If working	0.094*** [0.024]	0.029 [0.039]
Constant	1.089*** [0.171]	0.094 [0.437]

Observations	5696	1783
R–squared	0.27	0.34

Short–run price elasticity	−0.275	−0.135
Long–run price elasticity	−0.733	−0.356
Discount factor	n.a	4.54
Discount rate	n.a	3.54

Note: Robust standard errors in [brackets];

* significant at 10%;

**significant at 5%;

***significant at 1%. The short-run price elasticity is the coefficients estimates of cigarette price, *β_3_*; the long-run price elasticity is calculated using the expression *∂E*(*LnC_it_*)/ *∂E*(*LnP_it_*) = *β̂*_3_ /(1–*β̂*_1_ – *β̂*_2_); and the implied discount factor is *β_2_/β_1_* and the implied discount rate is *β_1_/β_2_*-1.
